# Observation Versus Embolization in Patients with Blunt Splenic Injury After Trauma: A Propensity Score Analysis

**DOI:** 10.1007/s00268-015-3387-8

**Published:** 2015-12-30

**Authors:** Dominique C. Olthof, Pieter Joosse, Patrick M.M. Bossuyt, Philippe P. de Rooij, Loek P. H. Leenen, Klaus W. Wendt, Frank W. Bloemers, J. Carel Goslings

**Affiliations:** Trauma Unit Department of Surgery, Academic Medical Center, Meibergdreef 9, 1105 AZ Amsterdam, The Netherlands; Surgical Department, Medisch Centrum Alkmaar, Alkmaar, The Netherlands; Clinical Research Unit, Academic Medical Center, Amsterdam, The Netherlands; Trauma Research Unit Department of Surgery, Erasmus MC, University Medical Center Rotterdam, Rotterdam, The Netherlands; Department of Surgery, University Medical Center Utrecht, Utrecht, The Netherlands; Department of Surgery, University Medical Center Groningen, Groningen, The Netherlands; Department of Surgery, VU University Medical Center, Amsterdam, The Netherlands

## Abstract

**Background:**

Non-operative management (NOM) is the standard of care in hemodynamically stable patients with blunt splenic injury after trauma. Splenic artery embolization (SAE) is reported to increase observation success rate. Studies demonstrating improved splenic salvage rates with SAE primarily compared SAE with historical controls. The aim of this study was to investigate whether SAE improves success rate compared to observation alone in contemporaneous patients with blunt splenic injury.

**Methods:**

We included adult patients with blunt splenic injury admitted to five Level 1 Trauma Centers between January 2009 and December 2012 and selected for NOM. Successful treatment was defined as splenic salvage and no splenic re-intervention. We calculated propensity scores, expressing the probability of undergoing SAE, using multivariable logistic regression and created five strata based on the quintiles of the propensity score distribution. A weighted relative risk (RR) was calculated across strata to express the chances of success with SAE.

**Results:**

Two hundred and six patients were included in the study. Treatment was successful in 180 patients: 134/146 (92 %) patients treated with observation and 48/57 (84 %) patients treated with SAE. The weighted RR for success with SAE was 1.17 (0.94–1.45); for complications, the weighted RR was 0.71 (0.41–1.22). The mean number of transfused blood products was 4.4 (SD 9.9) in the observation group versus 9.1 (SD 17.2) in the SAE group.

**Conclusions:**

After correction for confounders with propensity score stratification technique, there was no significant difference between embolization and observation alone with regard to successful treatment in patients with blunt splenic injury after trauma.

## Introduction

Trauma is a leading cause of mortality globally, especially among people below the age of 40 years [1, 2]. One of the organs frequently injured after blunt abdominal trauma is the spleen [3]. In the past, splenic injuries were treated with laparotomy and splenectomy. Nowadays, in hemodynamically (HD) stable patients without other indications for laparotomy, non-operative management (NOM) is the standard of care. NOM includes close observation of the patient and can be supplemented with splenic artery embolization (SAE).

SAE is generally reported to increase the success rate of NOM, approaching 98 % [4–10]. However, according to Harbrecht et al., those studies that demonstrated improved splenic salvage rates with SAE primarily compared SAE with historical NOM controls, as opposed to using contemporaneous controls or randomized controlled study designs [11]. In a second paper, looking at NOM in general (observation supplemented with SAE, if necessary), Harbrecht et al. showed that the improvement in the success rate of NOM of patients with blunt splenic injuries over time is caused, in part, by the increase in detection of relatively minor splenic injuries [12]. Thus, although SAE appears a promising strategy for improving successful treatment rates, its role should be further investigated, preferably in a well-designed prospective (randomized) controlled trial comparing it to strictly observational management.

 Such a trial, comparing observation to SAE, would require a large sample size (approximately 940 patients to detect 5 % difference in failure rate (i.e., the need for surgery)). Furthermore, it would be considered unethical to withhold SAE from a patient with, for example, a high-grade splenic injury or a contrast extravasation. However, previous research has shown that there is a need for such a randomized controlled trial [13]. Requarth et al. have addressed this issue in a meta-analysis [14]. The authors showed that SAE was associated with significantly lower failure rates in the higher grade splenic injuries (AAST grades 4 and 5). The available data did not enable the authors to look at contrast extravasation or the presence of pseudoaneurysm on CT and its relationship to the value of SAE, which is essential as the presence of these findings is a (possible) indication for SAE.

As an alternative, propensity scoring matching (PSM) analysis can be applied, a methodology that is used to control for treatment selection bias and to simulate, as closely as possible, the randomization process [15]. Using PSM analysis, we set out to investigate whether SAE improves success rate compared to observation alone in patients with blunt splenic injury.

## Materials and methods

### Study design

A retrospective, multicenter, cohort study was performed using the local Trauma Registries, a prospective, comprehensive registration of all acutely (within 24 h) admitted trauma patients. Five Level 1 Dutch trauma centers participated in the study. Data were collected from January 2009 to December 2012. All adult patients (age ≥ 16 years) with blunt splenic injury (Abbreviated Injury Scale codes 544299.2, 544210.2 through to 544228.5 and 544240.3, AIS manual Update 98 [16]) who were initially treated non-operatively were included. Initial treatment strategy was defined as the first selected treatment strategy following admission. Patients who were treated operatively, patients who died in the emergency department, and patients transferred from another hospital (unless findings of initial assessment were adequately documented) were excluded.

The Medical Research Involving Human Subjects Act (in Dutch: WMO) exempts this type of research from informed consent. The ethics committees of all five hospitals confirmed that official approval was not required.

### Outcome measures

The primary endpoint, successful treatment, was defined as the combination of splenic salvage without the need for a (re-)intervention. Treatment was considered unsuccessful if a splenectomy or another type of re-intervention was performed. Re-interventions included SAE or splenic surgery for patients who were initially selected for observation, and re-SAE or splenic surgery for patients who were initially embolized. Only re-interventions occurring within 30 days after discharge were taken into consideration. Secondary endpoints were (all-cause) complications and transfused blood products (defined as the total number of transfused packed cells, fresh frozen plasma, and thrombocytes during index admission). A complication was defined as any medical procedure performed for an undesirable event (whether spleen-related or not) during index admission (e.g., additional imaging, medication (e.g., antibiotics), drop in hemoglobin requiring transfusion).

### Data collection

Data collection was performed on location by one researcher (DO). The following data were collected: age, gender, Injury Severity Score (ISS), total length of hospital stay and length of stay in the Intensive Care Unit, systolic blood pressure (SBP), pulse rate, respiratory rate, Glasgow Coma Scale (GCS), intubation (yes or no), hemoglobin in g/dl, and imaging data. Splenic injury was diagnosed or confirmed by i.v. contrast-enhanced Computed Tomography (CT) scanning. The Organ Injury Scale of the American Association for the Surgery of Trauma (AAST) was used to grade splenic injury [17]. A contrast blush was defined as a well-circumscribed, peri-splenic or intraparenchymal contrast collection that was hyperdense with respect to the rest of the splenic parenchyma. The values that were used for SBP are the first values measured upon arrival at the emergency department. For transferred patients, the values (if known) and treatment strategy (if performed) in the hospital of initial assessment were described.

### Statistical analysis

Propensity scoring matching (PSM) is a methodology that can be used to adjust for treatment selection bias intrinsic to any observational study to simulate a randomization process as closely as possible [15]. In propensity score methods, balance on covariates (or confounders) is achieved through matching. Matching is based on the estimated chance of receiving the treatment or simply the propensity score (see Box 1 for more information about propensity score methods) [18].

Within each group of our study (observation and embolization), the proportion of participants with the endpoints defined above was calculated. Subsequently, the propensity score (probability of being treated with SAE instead of observation) for each of the individual patients was calculated, based on a multivariable logistic regression model including age, SBP, grade of splenic injury (grade 1–5 according to the AAST grading system), the presence of contrast extravasation on i.v. contrast-enhanced CT scanning, and ISS. The variables that were included in the logistic regression model had been identified in a previous systematic review [19]. The scale of the continuous variables was checked using fractional polynomials [20]. Subsequently, five strata were created based on the quintiles of the propensity score distribution in the cohort. Within each of the five strata, the relative risk (RR) was calculated. Across strata, a weighted relative risk was then calculated to express the chances of the respective endpoint with SAE compared to observation. Because the secondary endpoint of transfused blood products is a continuous variable, a linear regression model was built and covariate adjustment using the propensity score was performed to calculate the mean number of transfused blood products, conditional on the propensity score.

Data analysis was performed using IBM SPSS^®^ software package version 20 (Inc., Chicago, IL, USA), STATA version 11 (StataCorp. 2009. Stata Statistical Software: Release 11. College Station, TX: StataCorp LP), and SAS version 9.2. Relative risks and weighted relative risks were reported with their 95 % confidence interval. Categorical data are expressed as number (percentage) and continuous data as mean with standard deviation (SD) or median with interquartile ranges.
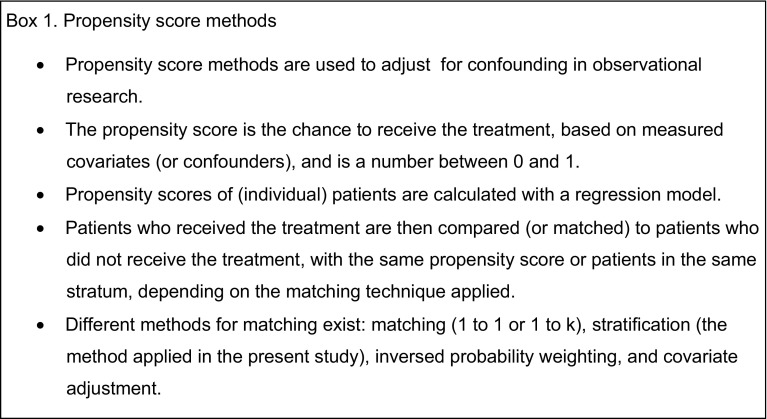


## Results

### Patients

Two hundred forty-seven patients were eligible for inclusion. Ten patients had to be excluded because they died of their injuries shortly after admittance to the Emergency Department. Twelve patients were excluded because the splenic injury was incorrectly coded as such or was not diagnosed before an emergency laparotomy. Nineteen other patients had to be excluded because they had been transferred from another hospital, and insufficient information was available about initial assessment and clinical course.

The study cohort consisted of 206 patients. Propensity scores ranged between 0.001 and 1.00. In three patients, the propensity score could not be calculated because, instead of a CT-scan with intravenous contrast, other imaging modalities had been performed (e.g., FAST). The participants were divided into five different strata with the following cut-offs for propensity scores: 0.007, 0.023, 0.126, and 0.75. Stratum 1 contains the patients with the highest propensity score (highest chance of receiving embolization based on the clinical parameters age, SBP, grade of splenic injury, the presence of contrast extravasation, and ISS) and stratum 5 the patients with the lowest propensity score. Table 1 displays baseline characteristics per stratum. The five groups of patients were balanced in terms of the variables used to calculate the propensity score, suggesting that the propensity stratification was adequate. The type of embolization was proximal in 36 of the 57 patients (63 %) and distal in 21 patients (37 %). In one patient, the contrast extravasation detected on the CT-scan could not be visualized during angiography, and the interventional radiologist refrained from embolization.

**Table 1:** Baseline characteristics of observed and embolized patients per stratum

	Observation (*n* = 149)	SAE (*n* = 57)
Age (years)		
Stratum 1	38 (25–45)	46 (32–59)
Stratum 2	42 (23–60)	30 (21–48)
Stratum 3	26 (19–40)	24 (21–42)
Stratum 4	47 (29–62)	–
Stratum 5	25 (19–41)	–
ISS (points)		
Stratum 1	33 (19–36)	33 (22–50)
Stratum 2	23 (14–39)	22 (16–29)
Stratum 3	21 (15–32)	26 (11–37)
Stratum 4	29 (20–35)	–
Stratum 5	20 (13–29)	–
SBP (RTS categories)*		
Stratum 1		
50–75	0 (0)	4 (11)
76–89	0 (0)	2 (6)
>89	5 (100)	29 (83)
Stratum 2		
76–89	2 (9)	2 (11)
>89	21 (91)	16 (89)
Stratum 3		
76–89	2 (5)	0 (0)
>89	35 (95)	4 (100)
Stratum 4		
76–89	1 (2)	–
>89	40 (98)	–
Stratum 5		
>89	40 (100)	–
Contrast extravasation		
Stratum 1		
Yes	5 (100)	33 (94)
No	0 (0)	2 (3)
Stratum 2		
Yes	15 (65)	17 (94)
No	8 (35)	1 (6)
Stratum 3		
Yes	1 (3)	0 (0)
No	36 (97)	4 (100)
Stratum 4		
Yes	0 (0)	–
No	41 (100)	–
Stratum 5		
Yes	0 (0)	–
No	40 (100)	–
Grade of splenic injury		
Stratum 1		
Grade 1	–	1 (3)
Grade 3	1 (20)	11 (31)
Grade 4	4 (80)	20 (57)
Grade 5	0 (0)	3 (9)
Stratum 2		
Grade 1	4 (17)	2 (11)
Grade 2	6 (26)	4 (22)
Grade 3	9 (39)	8 (44)
Grade 4	4 (17)	4 (22)
Stratum 3		
Grade 1	2 (5)	0 (0)
Grade 2	4 (11)	0 (0)
Grade 3	25 (68)	2 (50)
Grade 4	6 (16)	2 (50)
Stratum 4		
Grade 1	15 (37)	–
Grade 2	26 (63)	–
Stratum 5		
Grade 1	37(93)	–
Grade 2	3 (8)	–

Data are expressed as number (percentage) or median (interquartile range)

Patients with the highest propensity score are located in stratum 1 and patients with the lowest propensity score in stratum 5

*ISS* injury severity score, *SAE* splenic artery embolization, *SBP* systolic blood pressure

* Class I: 1–49, Class II: 50–75, Class III: 76–89, Class IV: >89 mmHg

A total of 14 patients died. Eight patients died due to traumatic brain injury, 4 patients died of persistent blood loss and uncontrollable hypotension because of shock in combination with multi-organ failure, one patient died after dislocation of the endovascular prosthesis of the superior mesenteric artery (SMA) following a traumatic aorta and SMA dissection, and one patient died on the IC unit after cardiopulmonary resuscitation (cardiogenic shock).

### Successful treatment

NOM was successful in 180 patients; in 134 (92 %) of the observed patients and in 48 (84 %) of the patients with SAE (Table 2). All embolized patients were in stratum 1, 2, or 3. After adjusting for age, grade of splenic injury, the presence of contrast extravasation, SBP, and ISS with propensity score stratification, there was no significant difference between the observed and the embolized patients in terms of successful treatment (weighted RR of 1.17 (0.94–1.45)). Table 2 depicts the RR per stratum.

**Table 2:** Successful treatment within the five strata

PS stratum	Observation *n* (%)	SAE *n* (%)	Relative risk
Stratum 1	3/5 (60)	28/35 (80)	1.33 (0.64–2.78)
Stratum 2	18/23 (78)	16/18 (89)	1.14 (0. 87–1.49)
Stratum 3	33/37 (89)	4/4 (100)	1.12 (1.00–1.25)
Stratum 4	40/41 (98)	–	–
Stratum 5	40/40 (100)	–	–
Overall	134/146* (92)	48/57 (84)	1.17 (0.94–1.45)

Patients with the highest propensity score are located in stratum 1 and patients with the lowest propensity score in stratum 5

*SAE* splenic artery embolization

* In three of the 149 patients, the propensity score could not be calculated; it was unknown whether a contrast extravasation was present

**Fig. 1:**
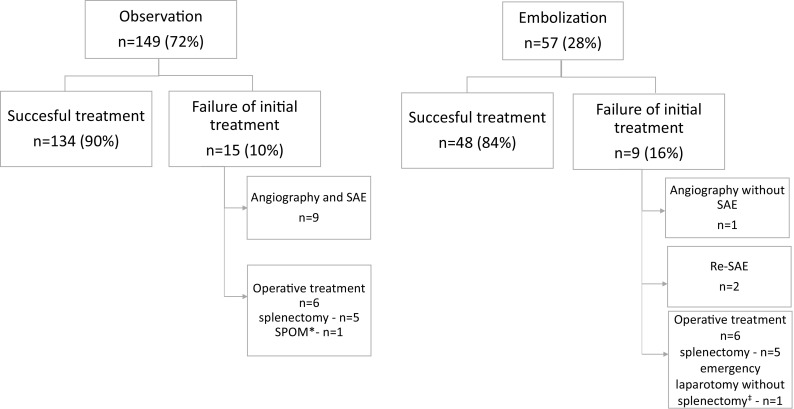
Failure of initial treatment and the type of re-interventions

Initial treatment failed in 24 patients (12 %) (Fig. 1). In total, five patients (2 %) were readmitted to hospital, of which one required a re-intervention. Two patients were treated with percutaneous drainage of a fluid collection surrounding the spleen (complication).

### Complications and transfused blood products

A total of 140 complications occurred in 89 patients. Table 3 displays the complications during index admission within the five strata. The overall weighted RR was 0.71 (0.41–1.22).

**Table 3:** Complications during index admission within the five strata

PS stratum	Observation *n* (%)	SAE *n* (%)	Relative risk
Stratum 1	3/5 (60)	17/35 (49)	0.81 (0.37–1.79)
Stratum 2	12/23 (52)	8/18 (44)	0.86 (0.45–1.63)
Stratum 3	15/37 (41)	0/4 (0)	0 (0–1.70)
Stratum 4	23/41 (56)	–	**–**
Stratum 5	10/40 (25)	–	**–**
Overall	63/146* (43)	25/57 (44)	0.71 (0.41–1.22)

Patients with the highest propensity score are located in stratum 1 and patients with the lowest propensity score in stratum 5

*SAE* splenic artery embolization

* In three of the 149 patients, the propensity score could not be calculated; it was unknown whether contrast extravasation was present

The most frequent complications were pulmonary-related (e.g., pneumonia or chest tube placement for pleural fluid (33/138; 23 %)). The second most frequently occurring complication was rebleeding (21/138; 15 %). All but one of the rebleeds were related to the splenic injury. In 32 (23 %) patients, spleen-related complications occurred. These included additional abdominal imaging because of suspected spleen-related clinical deterioration of the patient (*n* = 8), the development of a subphrenic abscess after splenectomy (*n* = 1), splenic pseudoaneurysm (*n*= 1), puncture site pseudoaneurysm (*n* = 1), and chronic cough after coiling (*n* = 1).

There was a trend toward a higher transfusion requirement for the embolized patients in the second stratum (Table 4), but overall there was no significant difference with regard to the total number of transfused blood products [mean of 4.4 (SD 9.9) in the observed vs. 9.1 (SD 17.2) in the embolized patients; *p* value 0.75].

**Table 4:** Mean number of transfused units of blood products within the five strata

PS stratum	Observation mean (SD)	SAE mean (SD)	*P* value
Stratum 1	1.2 (1.3)	9.8 (18.9)	0.32*
Stratum 2	1.5 (3.1)	9.2 (15.4)	0.053*
Stratum 3	3.0 (5.7)	1.8 (2.1)	0.68*
Stratum 4	7.1 (13.8)	–	–
Stratum 5	3.2 (7.6)	–	–
Overall (conditional on the Ps)	4.4 (9.9)	9.1 (17.2)	0.75^†^

Data are presented as mean (SD)

Patients with the highest propensity score are located in stratum 1 and patients with the lowest propensity score in stratum 5

*SAE* splenic artery embolization

* Independent T test

^†^ *P* value was calculated with a linear model and covariate adjustment using the propensity score

## Discussion

After correction for confounders with a propensity score stratification technique, there was no significant difference between SAE and observation alone with regard to successful treatment, all-cause complications, and transfusion requirements of patients with blunt splenic injury after trauma. The overall success rates for observation alone and SAE were 92 and 84 %, respectively.

To our knowledge, this is the first study comparing the successful treatment rate of SAE to that of observational management in contemporaneous patients. Observational management and SAE are very different non-operative management (NOM) modalities, and important information may be lost when the two modalities are studied together [14]. In particular, when the outcomes of patients treated with observational management and SAE are combined in NOM studies, the effectiveness of SAE may be masked. Harbrecht et al. state that the increasing use of CT scanning has resulted in an increase in the diagnosis of splenic injuries, and conclude that the proportionally greater numbers of moderately severely injured patients (ICD-9-CM 865.02) have also contributed to improved success rates of NOM over time [12]. However, although not significant, our results suggest (point estimates >1, in the advantage of embolisation) that the improvement in success might be attributed to the use of SAE.

All embolized patients were situated in stratums 1–3, leaving stratum 4 and 5 (lowest probability of undergoing SAE) empty. This is not surprising, as in daily practice, embolization is reserved for patients with higher splenic injury grade, the presence of a contrast extravasation, etc. The propensity score stratification technique allowed us to create ‘comparable strata,’ based on the risk factors for failure of observation identified in the literature, and to calculate the relative risk for success, complications, and transfusion requirement with SAE compared to observation within these strata. With the small overall differences in successful treatment rates for observation and embolization, and the lack of well-defined criteria for embolization (e.g., where to place the cut-off with regard to size of the blush), this propensity score matching analysis is an adequate method for analyzing the data. In addition, a propensity score matching analysis is a good alternative in a field where it is unimaginable that an RCT will be performed since embolisation has become an established treatment in the care for patients with splenic trauma.

In the literature, NOM is often declared to be successful if the spleen is salvaged. We used a combined endpoint (splenic salvage without re-intervention) because, with the increasing use of non-operative management, we found splenic salvage alone to be too crude a measure. Although splenic salvage is the most important outcome considering the lifelong risk of severe infection, differences exists between splenic salvage achieved after initial treatment and splenic salvage in which several re-interventions were necessary (from a patient-related point of view, due to use of resources and length of hospital stay). Therefore, we included re-interventions in the definition of failure of treatment.

The failure rate of NOM in our study is comparable to failure rates cited in the literature [9, 13, 21, 22] and the percentage of patients undergoing a re-intervention was equivalent in the NOM group (11 %) and the SAE group (16 %). While the majority of the patients in whom NOM failed underwent another non-operative attempt (SAE), two thirds of the embolized patients in whom treatment failed underwent operative management. This finding might support a more liberal (first) attempt with observation. However, it should be noted that patients with active bleeding who are hemodynamically compromised are not eligible for observation and should always undergo an intervention (six patients in our cohort).

The largest controversy regarding the optimal treatment strategy seems to be located in stratum 2, since the number of observed and embolized patients in this stratum is well-balanced. Further work, preferably with a prospective study design, needs to be done in this specific patient group to establish the best treatment modality.

A number of limitations of this study have to be considered. First, the presence of a contrast extravasation is thought to be one of the most important indications for embolization [6, 23–25]. This concept has recently been challenged by both Thompson and Michailidou et al., who demonstrated that a contrast blush is not an absolute indication for an operative or angiographic intervention, but that it is the size of the blush that matters. Thompson et al. identified a size of >1 cm as an important element predicting the need for intervention [26], while Michailidou et al. defined a cut-off value of 1.5 cm or greater diameter [27]. However, we did not assess the location of the contrast extravasation (intraparenchymal or contained vs. intraperitoneal or free) or the size of the contrast extravasation. This is a limitation, as it might be that large blushes are overrepresented in one group, thereby possibly introducing bias. Although it was standard policy to perform SAE in the presence of a contrast extravasation, the majority of the hospitals recruiting patients to the study do not employ a strict protocol. Differing protocols might have introduced bias for which we did not correct with the propensity analysis and this might have weakened the validity of the study. Another limitation, intrinsic to the propensity analysis, is that we could not control for unmeasured confounders. Recently, published studies conclude that SAE improves the success rate of NOM for grade 4 and 5 injuries [14, 28, 29]. Although we had a significant number of patients with grade 4 splenic injury, only 3 patients suffered from grade 5 injury. The validity of the results is therefore limited in this patient group. Lastly, the present study was limited by the relatively low sample size and specifically the low number of patients in the different treatment groups in some strata (e.g., number of observed patients in stratum 1). The fact that the advantage of embolisation over observation could not be expressed in a statistically significant difference may be explained by the relatively small size and the associated lack of power. Future research with larger patient cohorts, for example, by means of (international) research cooperation and data sharing (e.g., individual patient data) will provide a more definitive answer.

## Conclusion

After correction for confounders with propensity score stratification technique, there was no significant difference between embolization and observation alone with regard to successful treatment in patients with blunt splenic injury after trauma.
